# Preparation and Characterization of a Novel Mucoadhesive Carvedilol Nanosponge: A Promising Platform for Buccal Anti-Hypertensive Delivery

**DOI:** 10.3390/gels8040235

**Published:** 2022-04-11

**Authors:** El-Sayed Khafagy, Amr S. Abu Lila, Nahed Mohamed Sallam, Rania Abdel-Basset Sanad, Mahgoub Mohamed Ahmed, Mamdouh Mostafa Ghorab, Hadil Faris Alotaibi, Ahmed Alalaiwe, Mohammed F. Aldawsari, Saad M. Alshahrani, Abdullah Alshetaili, Bjad K. Almutairy, Ahmed Al Saqr, Shadeed Gad

**Affiliations:** 1 Department of Pharmaceutics, College of Pharmacy, Prince Sattam Bin Abdulaziz University, Al-Kharj 11942, Saudi Arabia; a.alalaiwe@psau.edu.sa (A.A.); moh.aldawsari@psau.edu.sa (M.F.A.); sm.alshahrani@psau.edu.sa (S.M.A.); a.alshetaili@psau.edu.sa (A.A.); b.almutairy@psau.edu.sa (B.K.A.); a.alsaqr@psau.edu.sa (A.A.S.); 2 Department of Pharmaceutics and Industrial Pharmacy, Faculty of Pharmacy, Suez Canal University, Ismailia 41522, Egypt; mghorab@hotmail.com (M.M.G.); shaded_abdelrahman@pharm.suez.edu.eg (S.G.); 3 Department of Pharmaceutics and Industrial Pharmacy, Faculty of Pharmacy, Zagazig University, Zagazig 44519, Egypt; a.abulila@uoh.edu.sa; 4 Department of Pharmaceutics, College of Pharmacy, University of Hail, Hail 81442, Saudi Arabia; 5 Department of Pharmaceutics, National Organization for Drug Control and Research (NODCAR), Giza 12553, Egypt; n_mohamed_80@yahoo.com (N.M.S.); sanadrania3@yahoo.com (R.A.-B.S.); 6 Department of Molecular Drug Evaluation, National Organization for Drug Control and Research (NODCAR), Giza 12553, Egypt; mahgouba3@gmail.com; 7 Department of Pharmaceutical Sciences, College of Pharmacy, Princess Nourah Bint Abdul Rahman University, P.O. Box 84428, Riyadh 11671, Saudi Arabia; hfalotaibi@pnu.edu.sa

**Keywords:** bilosomes, buccal mucosa, carvedilol, heart biomarkers, mucoadhesive nanosponge

## Abstract

Carvedilol (CRV) is a non-selective third generation beta-blocker used to treat hypertension, congestive heart failure and angina pectoris. Oral administration of CRV showed poor bioavailability (25%), which might be ascribed to its extensive first-pass metabolism. Buccal delivery is known to boost drugs bioavailability. The aim of this study is to investigate the efficacy of bilosomes-based mucoadhesive carvedilol nanosponge for enhancing the oral bioavailability of CRV. The bilosomes were prepared, optimized and characterized for particle size, surface morphology, encapsulation efficiency and ex-vivo permeation studies. Then, the optimized formula was incorporated into a carboxymethyl cellulose/hydroxypropyl cellulose (CMC/HPC) composite mixture to obtain buccal nanosponge enriched with CRV bilosomes. The optimized bilosome formula (BLS9), showing minimum vesicle size, maximum entrapment, and highest cumulative in vitro release, exhibited a spherical shape with 217.2 nm in diameter, 87.13% entrapment efficiency, and sustained drug release for up to 24 h. In addition, ex-vivo drug permeation across sheep buccal mucosa revealed enhanced drug permeation with bilosomal formulations, compared to aqueous drug suspension. Consecutively, BLS9 was incorporated in a CMC/HPC gel and lyophilized for 24 h to obtain bilosomal nanosponge to enhance CRV buccal delivery. Morphological analysis of the prepared nanosponge revealed improved swelling with a porosity of 67.58%. The in vivo assessment of rats indicated that CRV-loaded nanosponge efficiently enhanced systolic/diastolic blood pressure, decreased elevated oxidative stress, improved lipid profile and exhibited a potent cardio-protective effect. Collectively, bilosomal nanosponge might represent a plausible nanovehicle for buccal delivery of CRV for effective management of hypertension.

## 1. Introduction

Compared to other routes of administration, the oral route represents the most favored, convenient, and extensively used route of administration since it provides benefits such as patient compliance, painless administration, etc. Nevertheless, the limited oral absorption, and consequently, the low oral bioavailability, due to weak water solubility, low membrane permeability, and/or P-glycoprotein efflux, pose a hurdle against the development of new chemical entities for oral administration [1].

Carvedilol (CAR) is a non-selective third generation beta-blocker used to treat hypertension, congestive heart failure and angina pectoris [2]. Carvedilol is practically water insoluble; with a solubility value of 4.4 μg/mL in water, and it has a weak basic character (pKa value 6.8) [3]. Oral administration of CRV showed poor oral bioavailability (≈25%) due to high exposure to first-pass metabolism [4]. Because of its high lipophilicity and limited water solubility, it is classified as a class II in the biopharmaceutical classification system. Several solubility-improving approaches, including micronization, inclusion complexation and solid dispersion, have been adopted to improve the aqueous solubility of CRV and thereby enhance its oral bioavailability [5,6,7,8]. Nevertheless, because of the limitation of these conventional dosage forms, these approaches have shown modest improvement in therapeutic efficacy.

Nanotechnology-based drug delivery systems are considered to be a viable strategy for improving the therapeutic efficacy of such lipophilic drugs [9,10]. Recently, various nano-systems have been reported to enhance lipophilic drugs’ solubility, bioavailability, and stability through lipid-based formulations [11,12,13]. Among them, liposomes and niosomes, showed the efficacy to entrap both lipophilic and hydrophilic drugs [14], and to protect the encapsulated drug from degradation by the gastrointestinal tract (GIT) enzymes to some extent [15,16]. However, drug leakage and storage instability are identified as the primary disadvantages of conventional nano-vesicular carriers. Bile salt stabilized nano-vesicular systems, known as bilosomes, have been recently introduced as an alternative to the commonly used conventional nano-vesicular carriers (liposomes and niosomes). Bilosomes are novel vesicular nanocarriers in which bile salts are incorporated into the lipid bilayers of conventional nano-vesicular systems. Because of their configuration, they show a higher resistance to disruption by gastric secretion along with higher storage stability, compared to conventional nano-vesicular systems [17,18,19]. Accordingly, encapsulating a lipophilic drug such as CRV into bilosomes might offer a viable mean for enhancing drug aqueous solubility and thereby its systemic bioavailability.

Buccal cavity has been widely tested as an alternative to the oral route as a site for drug administration [20]. Systemic drug delivery via the buccal route has several advantages over oral administration, such as avoiding the first-pass metabolism and avoiding pre-systemic elimination within the gastrointestinal tract [21]. Recently, a large variety of dosage forms have been developed and commercialized (tablets, gels, films, etc.) for buccal administration [22,23,24]. Among these systems, mucoadhesive sponges have shown the advantages of keeping their swollen gel structure for a prolonged time, allowing for a longer residence time and more efficient medication absorption. In addition, by incorporating a nano-vesicular system, such as bilosomes, in a sponge to form a nanosponge, the developed system can combine the advantages of both platforms and, thus, opens the door to more enhanced buccal drug delivery with unique advances such as improved localization in the buccal mucosa, minimized burst release, and controlled drug release [25,26].

The aim of this study was to explore the efficacy of mucoadhesive cellulosic derivative sponges loaded with CRV bilosomes (CRV BL-SP) as an innovative anti-hypertensive dosage form. The in vivo efficacy of such an innovative formula was assessed via pharmacodynamic evaluation of specific biochemical parameters as lipid profile (cholesterol, triglycerides, LDL, and HDL), oxidative stress parameters (ROS, NO, TAC) in the heart, compared to the commercially available drug.

## 2. Results and Discussion

### 2.1. Effect of Formulation Variables on Physicochemical Characteristics of CRV-Loaded Bilosomes

#### 2.1.1. Effect on Vesicle Size, Polydispersity and Zeta Potential

The mean size, PDI, and ZP of the prepared CRV-loaded bilosomes are summarized in Table 1. The vesicular size of different CRV-loaded bilosomes ranged from 217.2 ± 2.0 nm (BLS9) to 311.5 ± 4.3 (BLS2), depending on the effect of both lipid concentration and cholesterol concentration used in preparing different bilosome formulations. At fixed cholesterol concentrations, increasing lipid concentration from 1 to 3% significantly produced smaller bilosomes (*p* < 0.0001) (Table 1). The mean particle size of BLS9 prepared at 3% lipid concentration (217.2 ± 2 nm) was significantly lower than that of BLS6 (235.1 ± 5.1 nm) or BLS3 (252.8 ± 5.1 nm), prepared at 2 and 1% lipid concentration, respectively. This finding might be attributed to the availability of a greater surface area at a higher lipid level, leading to the incorporation of more CRV into the vesicle bilayer, which in turn could improve vesicular membrane packing, and consequently result in a significant decrease in vesicles size [27]. Similar findings were reported by El Menshawe et al., who demonstrated the negative impact of lipid concentration on the vesicle size of terbutaline-loaded bilosomes [28].

Similarly, incorporation of cholesterol within bilosomal membrane was found to significantly affect the vesicular size of CRV-loaded bilosomes. At fixed lipid concentrations, incorporating cholesterol within the bilosomal membrane resulted in a significant decrease in particle size. The particle size of BLS3, prepared at cholesterol concentration of 30%, was significantly lower than that of BLS1 lacking cholesterol (252.8 ± 5.1 vs. 311.5 ± 4.3 nm, respectively). Furthermore, increasing cholesterol concentration from 10 to 30% was found to reduce the particle size of CRV-loaded bilosomes. The particle size of BLS3, prepared with 30% cholesterol (252.8 ± 5.1 nm), was smaller than that of BLS2 (271.2 ± 5.8 nm), prepared with 10% cholesterol (Table 1). This effect might be ascribed to the membrane rigidizing effect of cholesterol, which makes the vesicle bilayer more compact. These results are inconsistent with those of Abdel Rahman et al. [29] who reported that cholesterol concentration had a significant impact on reducing the particle size of tretinoin-loaded phospholipid vesicles.

The polydispersity index (PDI) of any colloidal system is a measure of its size distribution. It is regarded as an index that might indicate the homogeneity or heterogeneity of the produced colloidal system [30]. Generally, low PDI values refer to a homogenous system. In this study, all CRV-loaded bilosomes had PDI values ranging from 0.18 to 0.67 (Table 1), which may be interpreted as an acceptable mid-range showing a good size distribution and assuming homogeneity of the formulations.

The zeta potential is a key indicator of the colloidal stability of bilosomal formulations. Generally, a zeta potential value ±20 mV is crucial for colloidal stability due to electrical repulsion between particles [30]. As depicted in Table 1, the zeta potential of all bilosomal formulations was in negative range (−26.1 ± 3.7 mV to −46.1 ± 1.1 mV) (Table 1). In addition, at a fixed cholesterol concentration, a significant increase (*p* < 0.05) in the zeta potential values was observed upon by increasing phospholipid concentration from 1 to 3%.

#### 2.1.2. Effect on Percentage Entrapment Efficiency

Entrapment efficiency is regarded as a crucial criterion for assessing the quality of the fabricated bilosomes. As depicted in Table 1, the percentage entrapment efficiency of the formulated bilosomes fluctuated from 51.03 ± 0.5 to 87.13 ± 0.5%, depending on different formulation variables. Increasing lipid concentration from 1 to 3% remarkably elevated entrapment efficiency of bilosomes from 51.03 ± 0.5 (BLS1) to 79.57 ± 1.2 (BLS7) (Table 1). Such synergistic effect might be attributed to the higher surface area of lipid bilayer vesicles obtained at higher lipid concentration, which could provide more lipophilic area for the lipophilic drug (carvedilol) to be entrapped, giving rise to increased entrapment efficiency. These findings are consistent with those of Hasan et al. [31], who highlighted the synergistic impact of increasing phospholipid content from 1 to 3% on mebeverine hydrochloride entrapment efficiency inside ethosomes.

In the same context, the entrapment efficiency of the prepared bilosomes increased as cholesterol concentration increased from 0 to 30%. At fixed lipid concentration, the entrapment efficiency of BLS1 and BLS3 formulations were 51.03 ± 0.5% and 77.05 ± 0.9%, respectively (Table 1). The positive effect of increasing cholesterol concentration on the entrapment efficiency might be ascribed, on the one hand, to cholesterol’s membrane stabilizing effect, which prevents the leakage of an entrapped drug outside the vesicles, and, on the other hand, to its ability to increase the hydrophobicity of bilosomal phospholipid bilayer, and thus, allowing higher entrapment of the lipophilic drug, carvedilol, within bilosomal vesicles [29].

#### 2.1.3. Effect on In Vitro Drug Release

**Figure 1:**
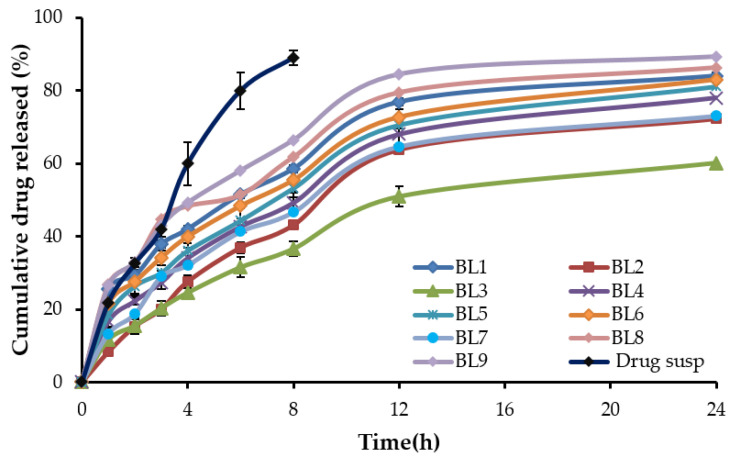
In vitro release profile of different CRV-loaded bilosomal formulations and CRV aqueous suspension. Data represents mean ± SD.

In vitro drug release pattern of CRV-loaded bilosomes, as well as free drug, are depicted in Figure 1. As shown in Figure 1, the rate of drug released from free CRV suspension was significantly higher than the investigated bilosomal formulations (*p* < 0.05). In addition, all bilosomal formulations exhibited biphasic release profiles with a rapid initial drug release in the first 3 h (ranging from 20.03 to 44.67%), followed by a sustained release profile till 24 h. The cumulative percentage release of CRV from bilosomes ranged from 60.12% (BLS3) to 89.3% (BLS9) after 24 h. The initial fast release of CRV from bilosomal formulation might be attributed to the presence of drug in the outer shell of the bilosome vesicles [32]. The impact of different formulation variables—namely, lipid concentration and cholesterol concentration—on the in vitro drug release from formulated bilosomes was also investigated. It was obvious that increasing lipid concentrations had a positive effect on the percentage of drug release from BLS. This effect might be ascribed, on the one hand, to the amphiphilic properties of the soya bean phosphatidylcholine, and on the other hand, to the negative effect of increasing lipid concentration on the vesicle size. In contrast, cholesterol concentration did not significantly affect the in vitro drug release pattern from the prepared bilosomal formulations (*p* = 0.894).

Analysis of release data, fitted into different release kinetic models (Table S1), revealed that CRV release from different bilosomal formulations followed the Higuchi kinetics, illustrating a diffusion-controlled mechanism [33], whilst free CRV suspension followed first-order kinetics.

### 2.2. Selection of Optimized Formula

Particle size, entrapment efficiency percentage and in vitro drug release from a vesicular carrier represent key parameters that might affect the in vivo behavior of the entrapped drug. In this study, therefore, the optimized formula was selected to have the least PS with maximum entrapment efficiency and maximum cumulative percent of CRV released after 24 h. Under such constrains, BLS9, containing lipid concentration (3%) and cholesterol (30% *w*/*w*), was chosen as the optimum formula among all other formulations for further investigations.

### 2.3. Characterization of the Optimized CRV-Loaded Bilosomes

#### 2.3.1. Transmission Electron Microscopic (TEM) Analysis

**Figure 2:**
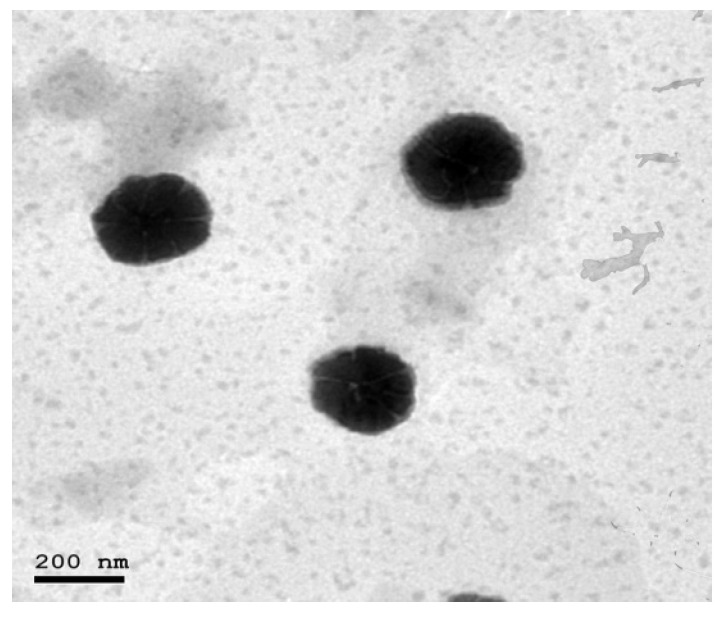
Transmission electron image of optimized bilosome formulation (BLS9).

The TEM micrograph of the optimized bilosome formulation (BLS9) is shown in Figure 2. The optimized bilosome was depicted in well-identified small unilamellar vesicles with a spherical shape. No accumulation or drug crystals were detected.

#### 2.3.2. Differential Scanning Calorimetry

**Figure 3:**
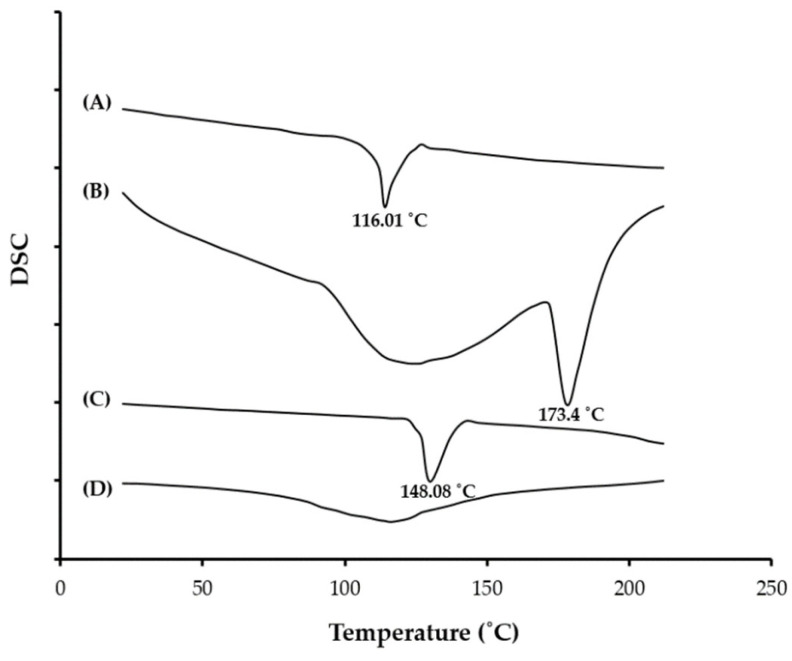
DSC thermograms of (A) pure carvedilol; (B) Soybean phosphatidylcholine; (C) Cholesterol and (D) optimized bliosomal formulation (BLS9).

The DSC thermograms of pure CRV, soybean phosphatidylcholine (SPC), cholesterol, and lyophilized optimized bilosomes (BLS9) formula are represented in Figure 3. The DSC thermogram of pure CRV unveiled a melting endothermic peak at 116 °C, corresponding to its melting point. Soybean phosphatidylcholine (SPC) and cholesterol exhibited endothermic peaks at 173.4 °C and 148.08 °C, respectively. Of interest, no endothermal peaks were observed in the thermogram of the lyophilized optimized bilosome formulation (BLS9), signifying that the drug is completely entrapped within bilosomal vesicles.

### 2.4. Ex-Vivo Release Study

#### 2.4.1. Ex-Vivo Permeation Studies

**Figure 4:**
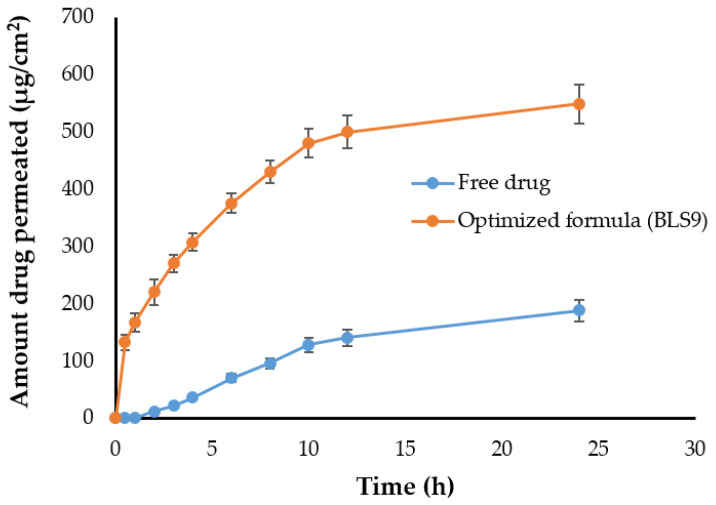
Ex-vivo permeation of CRV-loaded liposomes from sheep buccal mucosa. The data are represented as mean ± SD (*n* = 3).

The ex-vivo permeation study of CRV from optimized bilosomal vesicles and CRV aqueous suspension was conducted using sheep buccal mucosa. As shown in Figure 4, encapsulation of CRV in bilosome vesicles leads to a significant permeation improvement (*p* < 0.05) across the mucosal membrane relative to the control CRV suspension with a three-fold increase in the transdermal flux (J_max_). J_max_ of optimized bilosomal formulation was 22.82 ± 0.41 μg/cm^2^/h across sheep buccal mucosa, as compared to CRV aqueous suspension (7.83 ± 0.11 μg/cm^2^/h). This significant increase in transdermal flux of bilosomes might be attributed, on the one hand, to the small vesiclular size of CRV-loaded bilosomes, along with high phospholipids content, which increases the affinity of bilosomes to the biological membranes, thus increasing its permeation [34], and on the other hand, to the existence of bile salt that might help the permeation of the drug by opening the tight junction of the membrane [35]. Various permeation parameters through sheep buccal mucosa, were calculated and summarized in Table 2.

#### 2.4.2. Confocal Laser Scanning Microscopy (CLSM) Study

**Figure 5:**
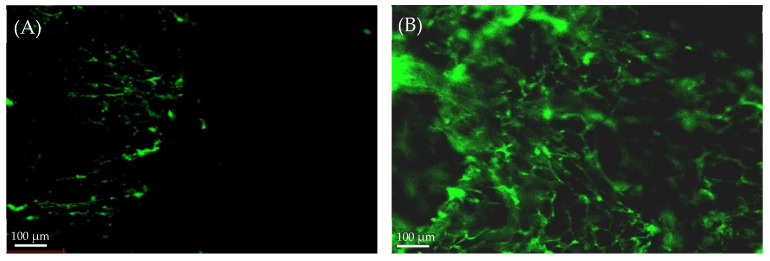
Confocal images of cross-sections of buccal mucosa after application of (**A**) rhodamine solution and (**B**) rhodamine-loaded bilosomes.

Confocal laser scanning microscopy (CLSM) is a valuable image tool to study the localization of bilosomes in the mucosal membrane. As illustrated in Figure 5, compared to rhodamine B solution, Rh-B loaded bilosomes showed deep penetration across the mucosal membrane, as manifested by higher great fluorescence intensity and homogeneous distribution. This deep penetration of bilosomes might be ascribed to the small vesicle size, which bestow a remarkably higher surface area. In addition, the presence of bile salts might help in enhancing drug penetration through the mucosal membrane [36]. Similar findings were reported by Saifi et al. [33] who demonstrated that entrapment of the antiviral, acyclovir, within bilosomal vesicles, could efficiently enhance drug penetration through intestinal mucosa, resulting in improved drug oral bioavailability.

### 2.5. Stability Study

After three months of storage at 4 ± 1 °C, there was no apparent alteration in the appearance of the optimized bilosome dispersion (BLS9). In addition, no remarkable differences were observed (*p* > 0.05) in the EE% and PS of the stored bilosomes compared to the fresh ones. The stored samples showed EE% of 84.5 ± 1.25% and particle size of 229.4 ± 2.65 nm; compared to EE% of 87.13 ± 0.5% and particle size of 217.2 ± 2 nm for fresh samples. These results clearly indicate the physical stability of bilosomal dispersion under the storage condition.

### 2.6. Development of CRV-Loaded Bilosomal Sponge (CRV Nanosponge)

CRV bilosomes loaded sponge (CRV nanosponge) and non-vesicular CRV sponge were successfully prepared in a matrix of carboxymethyl cellulose/hydroxypropyl cellulose (CMC/HPC) by a solvent casting method. The lyophilized sponge had a smooth surface texture with a porous structure. The porous structure of the sponge is created after water removal during lyophilization.

### 2.7. Characterization of CRV Nanosponge

#### 2.7.1. Sponge Morphology

Scanning electron microscopy (SEM) was adopted to examine the surface morphology of the prepared CRV nanosponge. SEM micrographs of CRV nanosponge illustrate a porous structure (Figure S1). This porous structure has resulted from the lyophilization process. The removal of water from bilosomal dispersion incorporated into the sponge by lyophilization created the vacant spaces, contributing to the recognized large number of uniform pores [37].

#### 2.7.2. pH, Drug Content and Porosity

CRV nanosponge was evaluated for pH, drug content and porosity. The pH of the formulated CRV nanosponge was 6.5 ± 0.22, which is considered compatible with buccal mucosa [38], nullifying the possibility of buccal mucosal irritation upon administration. Drug content of the prepared CRV nanosponge was found to be 97.75 ± 0.92%, indicating efficient drug incorporation. The porosity of the sponges is a critical factor in determining fluid absorption capability, and mechanical performance. In this study, CRV nanosponge showed improved porosity (67.58% ± 1.64), compared to non-vesicular CRV sponge (47.49% ± 1.32). The higher porosity of bilosomal nanosponge might be attributed to the incorporation of drug-loaded bilosome vesicles in the dispersion form, which would produce a highly porous structure upon lyophilization [37].

#### 2.7.3. Swelling Ratio and Mucoadehison Time

The degree of swelling of bioadhesive polymers is an essential factor that significantly affects adhesion power. Generally, adhesion occurs shortly after the beginning of swelling, whereas uptake of water results in relaxation of the originally entangled or twisted polymer chains, resulting in exposure of polymer bioadhesive sites for bonding to occur. The higher the polymer’s swelling, the faster the formation of adhesive bonds, and the better the adhesion is, which in turn would allow sufficient time for the drug entrapped in the bilosomes to permeate the buccal mucosa, making buccal delivery ideal. The swelling ratio of CRV nanosponge in PBS solutions as a function of time is given in Figure S2. It is obvious that CRV nanosponge showed an increasing trend in the swelling ratio with prolonging the immersing time. The swelling ratio of the prepared sponge enriched with bilosomes increased from 1.45 to 3.68 upon immersion in PBS for 1 and 4 h, respectively. This swelling ratio range is considered adequate for buccal delivery, based on previous findings of Hazzah et al. [26] who formulated curcumin nanoparticle-loaded sponge for buccal delivery with swelling ratios fluctuating from 1 to 3.5.

#### 2.7.4. In Vitro Mucoadhesion Time

Mucoadhesion time is considered a key determinant of the efficacy of drugs intended for buccal administration. Prolonging the residence time of mucoadhesive drug delivery systems in the buccal cavity allows intensified contact with the epithelial barrier, resulting in enhanced drug absorption, and thereby improves drug bioavailability. In this study, no displacement or detachment of the nanosponge was observed over 3 h. This relatively long mucoadhesion time is considered suitable for contacting with buccal mucosa, helping to wet the dosage form with the mucosal substrate, and thus allowing efficient drug release from the formulation.

#### 2.7.5. In Vitro Release of CRV from CRV Nanosponge

**Figure 6:**
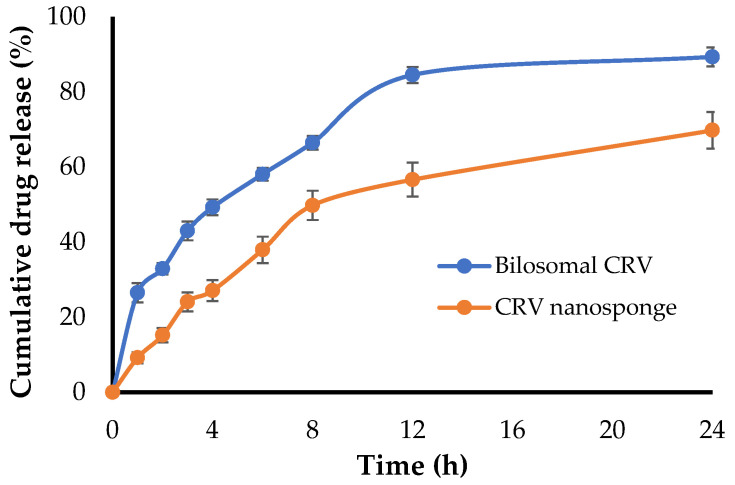
In vitro release profile of CRV from CRV-loaded bilosomes and CRV-loaded nanosponge. Data represents mean ± SD.

In vitro drug release pattern of CRV from either bilosomes or bilosomal sponge (nanosponge) are depicted in Figure 6. As shown in Figure 6, the in vitro release of CRV from BLS9 sponge after 24 h was much lower than that from bilosomes; the percent cumulative release of CRV from nanosponges and bilosomes were 69.77 ± 4.9% and 89.33% ± 2.5%, respectively. The slower drug release from nanosponge might be attributed to the fact that, upon sponge hydration, the polymer regains its gel structure, leading to an increase in diffusional path length for the drug, which consequently may delay the release [37].

### 2.8. In Vivo Studies of CRV Nanosponge

#### 2.8.1. Effect on Systolic and Diastolic Blood Pressure

**Figure 7:**
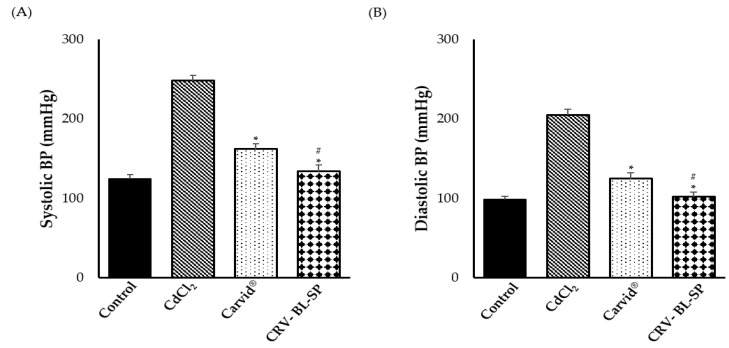
Effect of cadmium exposure and carvedilol bilosomes loaded sponge on (**A**) Systolic blood pressure and (**B**) Diastolic blood pressure. * *p* < 0.05 vs. CdCl_2_-intoxicated rats, ^#^ *p* < 0.05 vs. Carvid^®^-treated group.

In order to assess the pharmacological efficacy of the CRV nanosponge, the hypotensive potential of CRV was challenged against CdCl_2_-induced hypertension in rats. Hypertension was induced by intraperitoneal injection CdCl_2_ into rats for 14 days. Then, animals were treated with either CRV nanosponge or a commercially available marketed product (Carvid^®^, MULTI-Apex Pharma, Alexandria, Egypt) for two weeks. Finally, the systolic and diastolic blood pressures were recorded by the tail cuff method. As illustrated in Figure 7, intraperitoneal injection of CDCl_2_ for 14 consecutive days resulted in a significant increase in both systolic and diastolic blood pressure, compared to the negative control group. Treatment with either CRV nanosponge or commercially available marketed product (Carvid^®^) significantly lowered both systolic and diastolic blood pressure, compared to CdCl_2_-treated animals. Of interest, superior to Carvid^®^, CRV nanosponge was efficient in reducing both systolic and diastolic blood pressures to the normal control level. These results suggest the efficacy of CRV nanosponge in mitigating the blood pressure in hypertensive rats, to the near-normal control level.

#### 2.8.2. Effect on Cardiac Biomarkers

**Figure 8:**
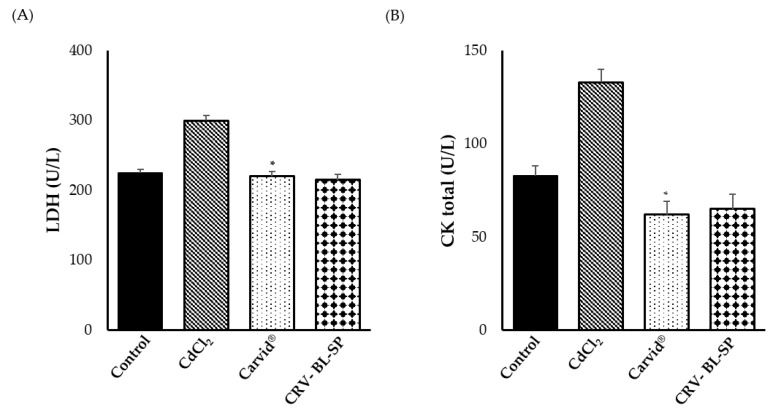
Effect of cadmium exposure and carvedilol bilosomes loaded sponge on cardiac biomarkers; (**A**) Serum lactate dehydrogenase (LDH); and (**B**) Serum creatine kinase (CK). * *p* < 0.05 vs. CdCl_2_-intoxicated rats.

Cadmium (Cd) is a potent cardiotoxic heavy metal that is reported to induce oxidative stress and membrane disturbances in cardiac myocytes [39]. Serum lactate dehydrogenase (LDH) and creatine kinase (CK) are widely used markers of tissue damage. These enzymes are released into the bloodstream from the heart, and they could reflect the alterations in myocardial membrane permeability. Accordingly, in the present study, we followed the changes of such markers in CdCl_2_-intoxicated rats and evaluated the protective effect of carvedilol. As shown in Figure 8, there was a marked elevation in serum LDH and CK activities for CdCl_2_-intoxicated rats, as compared to the control animals, indicating possible myocardial damage. In contrast, pretreatment with either CRV nanosponge or commercially available marketed product (Carvid^®^) could induce a significant (*p* < 0.05) reduction in the activities of the tested enzymes, compared to the CdCl_2_-intoxicated rats. Most importantly, CRV nanosponge could efficiently restore cardiac enzyme activities to their normal values as in the negative control group, which in turn, was beneficial in maintaining normal cardiac function. The plausible mechanism underlying the efficacy of CRV nanosponge on CdCl_2_-induced hypertensive rats might be linked to the powerful antioxidant activity of CRV, which restricts the leak of CK and LDH enzymes from myocardium tissue.

#### 2.8.3. Effect of CRV Nanosponge on Oxidative Stress Markers

**Figure 9:**
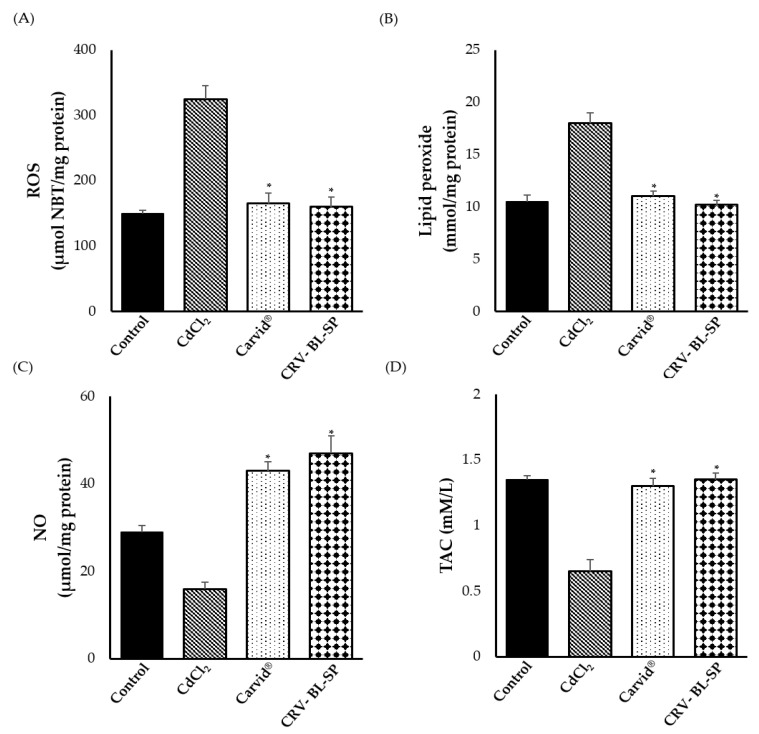
Effect of cadmium exposure and carvedilol bilosomes loaded sponge on oxidative stress markers levels; (**A**) reactive oxygen species (ROS); (**B**) lipid peroxidation (LPO); (**C**) nitric oxide (NO) and (**D**) total antioxidant capacity (TAC). * *p* < 0.05 vs. CdCl_2_-intoxicated rats.

Oxidative stress is a pivotal factor and key promoter of a number of cardiovascular diseases [40]. Accordingly, in this study we evaluated the efficacy of CRV nanosponge to alleviate oxidative stress in CdCl_2_-intoxicated rats. As shown in Figure 9, CdCl_2_ significantly elevated the levels of oxidative stress markers in the heart such as reactive oxygen species (ROS) and lipid peroxidation (LPO), and decreased nitric oxide (NO) and total antioxidant capacity (TAC) levels, compared to the control group, suggesting vascular endothelial cells dysfunction [41]. On the other hand, CRV nanosponge and commercially available marketed product (Carvid^®^) significantly (*p* < 0.05) decreased the heart contents of ROS and LPO while increasing the content of NO and TCA, compared to the CdCl_2_-treated animals (Figure 9). Cadmium has been reported to induce endothelial dysfunction via reducing NO• bioavailability [42]. Exposure to Cd causes an increase in O_2_^−^• production in the thoracic aorta, which could effectively scavenge NO to form strong oxidant peroxynitrite (ONOO^−^). Besides its action as a β-blocker, carvedilol has been demonstrated to have a potent antioxidant capacity. CRV exerts both ROS-scavenging and ROS-suppressive properties. In addition, it by preserves endothelium-derived nitric oxide activity. Lopez et al. [43] have emphasized the potential of carvedilol to protect against free-radical-induced endothelial dysfunction via by scavenging free radicals and enhancing the effect of NO. Accordingly, in this study, the efficient antioxidant activity of bliosomal CRV nanosponge might be ascribed to the reduced peroxynitrite formation in the endothelial cells and increase NO’s bioavailability through scavenging superoxide ions and inhibiting the production of superoxide radicals [44].

#### 2.8.4. Effect on the Serum Lipid Profile Parameters

**Figure 10:**
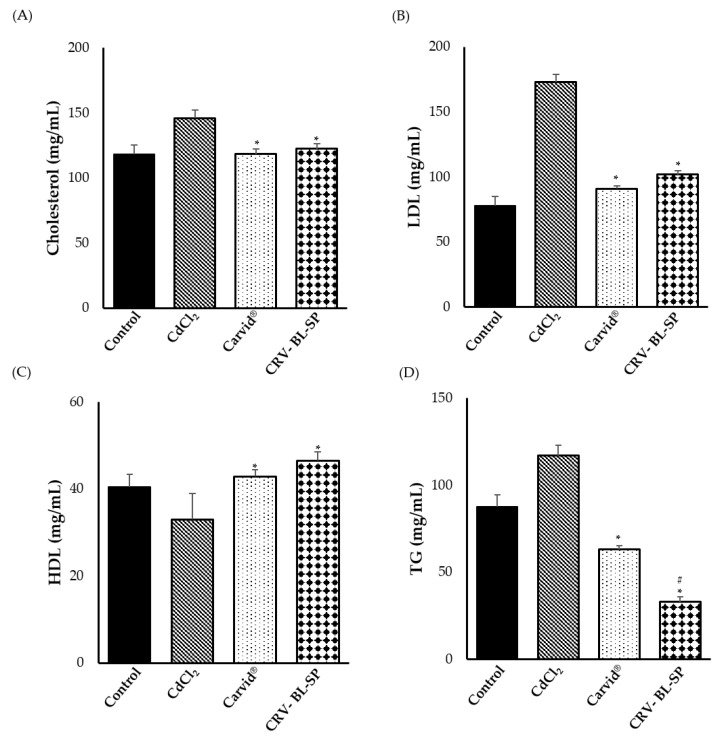
Effect of cadmium exposure and carvedilol bilosomes loaded sponge on lipid profile levels; (**A**) Cholesterol; (**B**) low density lipoproteins (LDL); (**C**) High density lipoproteins (HDL) and (**D**) triglycerides (TG). * *p* < 0.05 vs. CdCl_2_-intoxicated rats, ^#^ *p* < 0.05 vs. Carvid^®^-treated group.

Recent studies have revealed novel properties of carvedilol, which may function to protect the vasculature and heart chronic pathological conditions such as atherosclerosis. In the current study, therefore, the lipid profile (cholesterol, low density lipoprotein (LDL), high density lipoprotein (HDL) and triglycerides (TG)) was assessed in the serum of different treated groups. As shown in Figure 10, cholesterol, LDL, HDL and TG levels in serum showed significant elevations (*p* < 0.05) in the CdCl_2_-treated rats, compared to normal ones. On the contrary, treatment with either CRV nanosponge or commercially available marketed product (Carvid^®^) significantly reduced Cholesterol, LDL, and TG levels, while increasing the levels of HDL, compared to the CDCl2-treated group. The efficacy of CRV nanosponge in lessening the levels of cholesterol, TG, and LDL while increasing the levels of HDL, might be ascribed to the ability of carvedilol to inhibit the oxidation of LDL, preventing the creation of oxidized-LDL, which is thought to promote the development of atherosclerotic plaque [45], and thus protects vascular endothelial from damage.

### 2.9. Histopathological Examination

**Figure 11:**
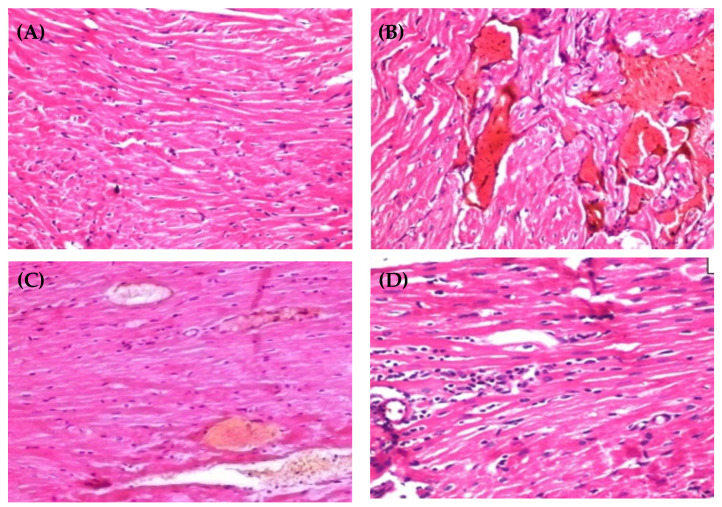
Histopathological sections of heart tissue of (**A**) control group, (**B**) CdCl2-intoxicated group, (**C**) Carvid^®^-treated group and (**D**) CRV nanosponge-treated group.

To gain an insight into the cardio-protective effect of different CRV formulations, heart tissues were collected from animals at the end of the experiment, and histological analysis was conducted (Figure 11). Obvious gross morphological anomalies were observed in myocardial cells of CdCl_2_-treated rats. Histological section of CdCl_2_-treated rats showed localized fatty changes in some myocardial cells and the presence of vascular blood gaps lined by damaged endothelium. In addition, there was edema with inflammatory cells in the pericardium. On the other hand, a micrograph of heart tissue of Carvid^®^-treated rats showed cardiac blood capillaries exhibiting moderate dilation. Of interest, histological sections in the heart tissues of animals treated with CRV nanosponge showed no remarkable histopathological alterations in myocardial cells. Collectively, histopathological studies indicated a superior cardio-protective effect of CRV nanosponge, compared to commercially available drugs (Carvid^®^).

## 3. Conclusions

In this study, CRV-bilosomes were developed and optimized. The optimized CRV-loaded bilosomes significantly increased buccal permeability as manifested by deeper penetration of drug-loaded bilosomes through sheep buccal mucosa. In addition, the optimized bilosomes formulation was successfully incorporated into a CMC/HPC sponge. The designed CMC/HPC bilosomal nanosponge showed acceptable characteristics such as pH and swelling ratio and a good residence time that allowed enough contact time for penetration. In addition, in vivo studies revealed that a CRV nanosponge could efficiently enhance systolic and diastolic blood pressure, decrease elevated oxidative stress, improve lipid profile and exhibit a potent cardio-protective effect. To sum up, bilosomal nanosponge might represent a promising carrier for buccal delivery of carvedilol for the management of hypertension with a superior cardio-protective effect, compared to the commercially available product (Carvid^®^).

## 4. Materials and Methods

### 4.1. Materials

Carvedilol (CRV) was provided by SAGA Pharmaceutical Company (Cairo, Egypt). Soybean phosphatidylcholine (SPC), cholesterol (CH), sodium deoxycholate (SDC), dialysis membrane (12,000–14,000 molecular weight cut-off), Griess reagent, thiobarbituric acid, carboxymethyl cellulose sodium salt (Na CMC; 50–200 cps) were purchased from Sigma Aldrich (St. Louis, MO, USA). Hydroxypropyl cellulose (HPC; 100,000 cps) was obtained from Fluka (Buchs, Switzerland). All other chemicals and reagents used were of analytical grade.

### 4.2. Preparation of Bilosomes (BLS)

CRV-loaded bilosomes were prepared by the thin-film hydration/sonication technique [46]. Briefly, accurately weighted amounts of Soybean phosphatidylcholine (SPC), cholesterol (Chol), diacetyl phosphate (DCP), and carvedilol (CRV) were completely dissolved in 5 mL dichloromethane. The resultant lipid solution was then evaporated at 60 °C under reduced pressure, using a rotary evaporator, to produce a thin lipid film. The lipid film was then hydrated using 25 mL distilled water containing 25 mg sodium deoxycholate (SDC). The formed CRV-loaded bilosomes were size-reduced by sonication (Crest UltraSonicator 575DAE, Trenton, NJ, USA) for 3 cycles of 5 min with an interval of 5 min. The obtained bilosome dispersions were stored in a refrigerator at 4 °C till further investigations. The composition of the prepared bilosomes is given in Table 1.

### 4.3. Characterization of CRV-Loaded Bilosomes

#### 4.3.1. Determination of Particle Size

A laser scattering particle size analyzer (Malvern Instrument Ltd., Worcestershire, UK) was used to determine particle size, polydispersity index, and zeta potential of the bilosomes. Before measurement, 100 μL of CRV-loaded bilosomes were suitably diluted (100 times) with distilled water. All measurements were conducted at 25 °C in triplicates.

#### 4.3.2. Transmission Electron Microscope (TEM)

The morphology of CRV-loaded bilosomes was detected by transmission electron microscope (Joel JEM 1230, Tokyo, Japan). Briefly, CRV-loaded bilosomes were diluted with distilled water and stained with phosphotungstic acid. The samples were then applied onto a carbon-coated copper gride and allowed to dry before being visualized by TEM at an accelerating voltage of 100 kV [47].

#### 4.3.3. Entrapment Efficiency (EE %) and Drug Loading (DL)

CRV entrapped in bilosomes was separated from the un-entrapped drug by centrifugation of bilosomal dispersion at 15,000 rpm for 0.5 h at 4 °C using a cooling centrifuge (Beckman, Fullerton, Canada). The collected supernatants were then analyzed spectrophotemetrically for CRV at λmax of 242 nm (Shimadzu, UV-1601 PC model, Kyoto, Japan) [48]. EE (%) and DL were calculated using the following equations [29].

$$\mathrm{EE}\;\left(\%\right)=\frac{\left(\mathrm{Di}-\mathrm{Dn}\right)}{\mathrm{Di}}\times 100$$

$$\mathrm{DL}=\frac{\left(\mathrm{Di}-\mathrm{Dn}\right)}{L}\times 100$$

where, Di is initial drug amount, Dn is the unentrapped drug, and L is total lipid concentration.

#### 4.3.4. Differential Scanning Calorimetry (DSC)

DSC thermograms of pure CRV, SPC, cholesterol, and the optimized lyophilized CRV-loaded bliosome formulation were recorded using Shimadzu differential scanning calorimeter (DSC-50, Kyoto, Japan). Samples (2 mg) were subjected to heat in the range of 10 to 300 °C at a constant heating rate of 10 °C/min in a standard aluminum pot under an inert nitrogen flow of 25 mL/min.

### 4.4. In Vitro Drug Release Study

The in vitro release study was conducted via a dialysis bag method using cellophane membrane (MW cut-off 12–14 KDa), which permits the diffusion of free drug but not lipid vesicles [49]. Briefly, adequate volume of CRV-loaded bilosomes, containing CRV equivalent to 6.25 mg, were placed in a cellophane bag suspended into 250 mL phosphate-buffered saline (PBS; pH 6.8) containing 0.5% sodium lauryl sulfate, and was maintained at 37 ± 1 °C and stirred at 50 rpm for 24 h. 1 mL samples were withdrawn at 1, 2, 3, 4, 5, 6, 8, 12 and 24 h, and was replenished with an equal volume of fresh buffer to maintain the sink conditions. The release of CRV from bilosomes was compared with CRV suspension. Drug concentrations in the samples were determined spectrophotometrically at 242 nm and cumulative drug release was estimated using the following equation:

$$\mathrm{Cumulative}\;\mathrm{drug}\;\mathrm{release}\;\left(\%\right)=\frac{\mathrm{Mt}}{\mathrm{Mi}}\times 100$$

where Mi is the initial amount of the CRV in bilosomes and Mt is the amount of drug released at time t.

To gain an insight into the mechanism of drug release from bilosomes, the results obtained from the release studies were kinetically analyzed for the order of drug release. The formulae were fitted to zero-order, first-order, and the Higuchi diffusion models, and various correlation coefficients (R2) were determined.

### 4.5. Ex-Vivo Permeation Study

In order to assess the ability of bilosomes to enhance drug permeation in vivo, the ex-vivo permeation of CRV-loaded bilosomes was investigated using sheep buccal mucosa. Briefly, sheep buccal mucosa was fixed at one end of a glass cylinder, while the other end of the cylinder was connected to the shafts of dissolution apparatus I (Figure S3). The permeation medium consisted of 250 mL phosphate buffer saline (PBS; pH 6.8) containing 0.5% sodium lauryl sulfate, and was maintained at 37 ± 1 °C and stirred at 50 rpm for 24 h. Adequate volume of CRV-loaded bilosomes, equivalent to 6.25 mg drug, was placed in the glass cylinders. At definite time points (0.5, 1, 2, 3, 4, 6, 8, 12, and 24 h), 1 mL aliquot samples were withdrawn from the permeation medium, followed by compensation with equal volumes of fresh medium. A similar experiment was conducted for CRV suspension, containing 6.25 mg drug. The amount of drug permeated was analyzed using a UV spectrophotometer (Shimadzu, Tokyo, Japan) at 242 nm. The cumulative amounts of drug permeated per unit area (μg/cm^2^) was graphically displayed as a function time, and the permeation parameters were determined. Drug flux at 24 h (Jmax) and enhancement ratio (ER) were calculated according to the following equations [31]:

$$\mathrm{Jmax}=\frac{\mathrm{Amount}\;\mathrm{of}\;\mathrm{drug}\;\mathrm{permeated}\;\mathrm{per}\;\mathrm{unit}\;\mathrm{area}}{\mathrm{Time}}$$

$$\mathrm{ER}=\frac{{\mathrm{Jmax}}_{\mathrm{Bilosomal}\;\mathrm{formulation}}}{{\mathrm{Jmax}}_{\mathrm{CRV}\;\mathrm{suspension}}}$$

Besides, the permeability coefficient C_p_ (cm/h) of CRV from bilosomes was calculated by dividing the slope of the curve by initial drug concentration.

### 4.6. Confocal Laser Scanning Microscopy (CLSM) Study

Confocal laser scanning microscopy (CLSM) was adopted to study the depth of penetration of the optimized bilosomes into the sheep buccal mucosa. Briefly, rhodamine B solution and rhodamine B-loaded bilosome formulation were applied to sheep buccal mucosa, and was then removed and washed with distilled water. The involved area was frozen at −20 °C and was then sectioned with a cryostat into 20 µm slices and put on slides and covered by glass coverslips. The slides were then microscopically inspected using an inverted laser scanning confocal microscope LSM 710 (CRVl Zeiss, Germany) equipped with an He/Ne laser operating at 524 nm for fluorescence excitation [33].

### 4.7. Stability Studies for Carvedilol-Loaded Bilosomes

The optimized CRV-loaded bilosomes preparation was tested for stability by keeping them in glass vials at 4 ± 1 °C for three months. Physical stability was evaluated by comparing the results of particle size and entrapment efficiency before and after storage time [46].

### 4.8. Preparation of Carvedilol Nanosponge

The optimized carvedilol-loaded bilosomal dispersion was added to carboxymethyl cellulose/hydroxypropyl cellulose (CMC/HPC) aqueous blend (1:1 ratio) and then stirred for 4 h to obtain a homogenous mixture. The resultant mixture was subsequently poured into a mold (1 cm in diameter), and lyophilized for 24 h.

### 4.9. Characterization of Carvedilol Nanosponge

#### 4.9.1. Sponge Morphology

The external surface of CRV-nanopsonge was observed by scanning electron microscopy (SEM, Quanta 250 FEG, FEI Co., Tokyo, Japan). The sponge was placed on double-sided adhesive carbon tape. The sponges were then sputter-coated with gold and set in a chamber of SEM and examined under a low vacuum at 200× magnification [26].

#### 4.9.2. Sponge pH

The surface pH of CRV-nanosponge was determined by a pH-meter (Jenway 3510, Barloworld Scientific, Stone, Staffordshire, UK). Briefly, one unit sponge (1 cm diameter) of CRV-nanosponge was allowed to swell in contact with 2 mL of simulated saliva fluid (pH 6.8) for 2 h at room temperature. Then the surface pH of the nanosponge was determined by bringing the electrode of the pH-meter into contact with the sponge surface and left to equilibrate for 1 min [50].

#### 4.9.3. Ex-Vivo Mucoadhesion Time

Sheep buccal mucosa was attached to the inner side of a beaker containing 100 mL simulated saliva fluid (SSF; pH 6.8), as depicted in Figure S4. One unit sponge (1 cm diameter) was moistened with 50 μL of SSF and then fixed to sheep buccal mucosa after applying a slight force. The simulated saliva fluid was stirred at a rate of 50 rpm and mucoadhesive time was recorded when a complete detachment of nanosponge occurred [50].

#### 4.9.4. Porosity Determination

Samples of known volume (V) and weight (Wi) were immersed in a graduated cylinder containing ethanol at 25 °C and soaked for 24 h [37]. The final wet sponge weight was recorded as Wf. Porosity (%) was calculated as follows:

Porosity (%) = ((Wf − Wi)/(ρ ethanol) ÷ V) × 100

where ρ ethanol: Density of ethanol

#### 4.9.5. Swelling Ratio

The sponge was initially weighed, then immersed in phosphate-buffered saline (PBS; pH 6.8). The sponge was them incubated at 37 °C for 1, 2, 3, and 4 h and reweighed after each time of incubation. The swelling ratio was estimated as follows [26]:

Swelling ratio = (Ww − Wi)/Wi

Wi is the sponge’s initial weight, and Ww is the sponge’s weight after hydration and removing the excess buffer.

#### 4.9.6. In Vitro Release of CRV Nanosponge

CRV-loaded nanosponge and bilosomal CRV, containing CRV equivalent to 6.25 mg, were placed in cellophane bag (cut-off 12–14 KDa) suspended into 250 mL phosphate buffer saline (PBS; pH 6.8) containing 0.5% sodium lauryl sulfate, and maintained at 37 ± 1 °C and stirred at 50 rpm. The cumulative % of CRV released was conducted for up to 24 h as previously discussed in Section 2.4.

### 4.10. In Vivo Studies

#### 4.10.1. Experimental Animals

Male Albino rats (180–200 g) were used for in vivo experiments. The study protocol was approved by the research ethics committee, Suez Canal University, Egypt (201804RA2). Rats were freely allowed to have food and water at a 12 h light/12 h dark cycle at room temperature (25 ± 1 °C).

#### 4.10.2. Experimental Protocol

Twenty-four male Albino rats (180–200 g) were categorized into 4 groups. The first group received 0.5% CMC orally and served as a normotensive control group. To induce hypertension in the remaining three groups, animals were intraperitoneally injected with cadmium chloride dissolved in 0.9% saline (1 mg/kg/day) for 14 days [51]. After the induction of hypertension, the animals were treated with either 0.5% CMC orally for 14 days (Group II), buccal administration of CRV-nanosponge (6.25 mg CRV/kg) for 14 days (Group III), or orally treated with a commercially available CRV product (6.25 mg CRV/kg) suspended in 0.5% CMC vehicle daily for another 14 days (Group IV). At the end of the experiment, rats were euthanized, and blood samples were collected without an anticoagulant. The separated serum was used for the assessment of lactate dehydrogenase [52] and creatine phosphokinase [53] activities using commercial enzymatic kits (Reactivos GPL, Barcelona, Spain). Cholesterol, LDL, HDL, and TG were also assessed. Total antioxidant capacity (TAC) was evaluated using a commercial kit (Biodiagnostic, Cairo, Egypt). All measurements were performed according to the manufacturer instructions.

#### 4.10.3. Heart Homogenate Isolation

The heart of each animal was excised instantly, washed with saline, and stored at −80 °C. A definite weight of the heart was then homogenized in chilled 50 mM phosphate-buffered saline (PBS; pH 7.4), centrifuged for 15 min at 1200 and 4 °C using a cooling centrifuge (Sigma 30K, Osterode, Germany). The supernatants were used for the detection of the oxidative stress parameters.

#### 4.10.4. Lipid Peroxidation Determination (LPO)

Lipid peroxidation (LPO) measurement of the heart was done by a colorimetric reaction with thiobarbituric acid as previousely described [54].

#### 4.10.5. Determination of Nitric Oxide (NO)

Nitric oxide was estimated as nitrite concentration. The technique used depends on the Griess reaction, which converts nitrite to a deep purple azo compound measured photometrically at 540 nm [55].

#### 4.10.6. Reactive Oxygen Species Determination (ROS)

This test measures the intracellular conversion of nitro blue tetrazolium (NBT) to formazan by superoxide anion, which was used to measure the production of reactive oxygen species (ROS) as previously described [56].

#### 4.10.7. Histopathological Examination

Autopsy heart samples were taken from different treated animals and fixed for 24 h in 10% formal saline. The samples were then washed with distilled water, followed by treatment with serial alcohol dilutions (methyl, ethyl, and absolute ethyl) for dehydration. Specimens were cleared in xylene and submerged for 24 h in hot air ovens at 56 °C. The tissues were examined by the electric light microscope.

### 4.11. Statistical Analysis

All experimental data were expressed as mean values ± S.D. Statistical analysis was performed using GraphPad Prism 8 (GraphPad Software, San Diego, CA, USA) adopting one-way analysis of variance (ANOVA). Post hoc testing was done for inter-group comparisons using Tukey’s multiple comparisons test. Differences were considered statistically significant at *p* ≤ 0.05.

## Figures and Tables

**Table 1:** Composition and physicochemical characteristics of CRV-loaded bilosomes.

Formulation Code	SPC Conc. (% *w*/*w*)	Chol Conc. (% *w*/*w*)	EE (%)	DL	PS (nm)	PDI	ZP (mV)
BLS1	1	0	51.03 ± 0.5	1.86 ± 0.04	311.5 ± 4.3	0.41 ± 0.005	−28.5 ± 3.0
BLS2	1	10	59.81 ± 1.4	2.15 ± 0.04	271.2 ± 5.8	0.54 ± 0.003	−26.1 ± 3.7
BLS3	1	30	77.05 ± 0.9	2.26 ± 0.03	252.8 ± 5.1	0.67 ± 0.004	−33.6 ± 1.0
BLS4	2	0	78.81 ± 1.5	1.10 ± 0.00	251.4 ± 3.4	0.36 ± 0.015	−34.8 ± 2.1
BLS5	2	10	82.8 ± 2.8	1.12 ± 0.04	241.3 ± 5.8	0.37 ± 0.006	−34.7 ± 2.9
BLS6	2	30	85.72 ± 1.4	1.15 ± 0.02	235.1 ± 5.1	0.21 ± 0.004	−38.1 ± 1.0
BLS7	3	0	79.57 ± 1.2	0.73 ± 0.01	231.3 ± 7.1	0.43 ± 0.003	−37.6 ± 1.9
BLS8	3	10	82.4 ± 1.8	0.75 ± 0.01	229.5 ± 6.0	0.43 ± 0.002	−43.3 ± 1.1
BLS9	3	30	87.13 ± 0.5	0.78 ± 0.02	217.2 ± 2.0	0.18 ± 0.002	−46.1 ± 1.1

Each formula contains 62.5 mg CRV, DCP (5% *w*/*w* of total lipid), and 25 mg SDC. EE, entrapment efficiency; DL, drug loading; PS, particle size, PDI, polydispersity index; ZP, zeta potential. Data represents mean ± SD of three independent experiments.

**Table 2:** Ex-vivo permeation parameters for CRV aqueous suspension and CRV-loaded bilosomes.

Permeation Parameters	CRV Aqueous Suspension	CRV-Loaded Bilosome
Total amount of drug permeated (μg/cm^2^)	188.0 ± 1.9	548.4 ± 6.9
J_max_ (μg/cm^2^/h)	7.83 ± 0.11	22.82 ± 0.41
ER (cm^−2^·h^−1^)	1	2.91
C_p_ (cm/h)	0.0017 ± 0.001	0.0071 ± 0.003

## Data Availability

Not applicable.

## Supplementary Materials

The following supporting information can be downloaded at: https://www.mdpi.com/article/10.3390/gels8040235/s1, Figure S1: Transmission electron image of CRV nanosponge; Figure S2: Swelling of CRV nanosponge; Figure S3: Cartoon depicting the apparatus utilized in ex-vivo permeation study of the optimized CRV-loaded bilosomes from Sheep buccal mucosa; Figure S4: Cartoon depicting the apparatus utilized in measuring the ex-vivo mucoadhesion time of the formulated BLS nanosponge; Table S1: Kinetic analysis of release data of carvedilol from different bilosomal formulations and aqueous CRV suspension.
