# Supplementary material for: Preparation and Characterization of a Novel Mucoadhesive Carvedilol Nanosponge: A Promising Platform for Buccal Anti-Hypertensive Delivery
Source: Gels. 2022 Apr 11;8(4):235. doi: 10.3390/gels8040235 (PMC9028337; doi:10.3390/gels8040235)
Supplement: Supplementary file 1 [file gels-08-00235-s001.zip › gels-1643962-supplementary.pdf]

Supplementary Materials

Table S1. Kinetic analysis of release data of carvedilol from different bilosomal formulations and aqueous CRV suspension.

Formulation code	Correlation Coefficient (R^2)		
	Zero-order	First-order	Higuchi model
BL1	0.8326	0.9302	0.9457
BL2	0.8362	0.9148	0.9472
BL3	0. 8791	0.9378	0.9583
BL4	0.9526	0.9512	0.9653
BL5	0. 8727	0. 9593	0.9667
BL6	0. 8533	0.9553	0.9605
BL7	0. 8481	0.9283	0.9558
BL8	0.8064	0. 9194	0. 0.9294
BL9	0.7774	0.9047	0.9184
Carvedilol suspension	0.9428	0.9879	0.9772

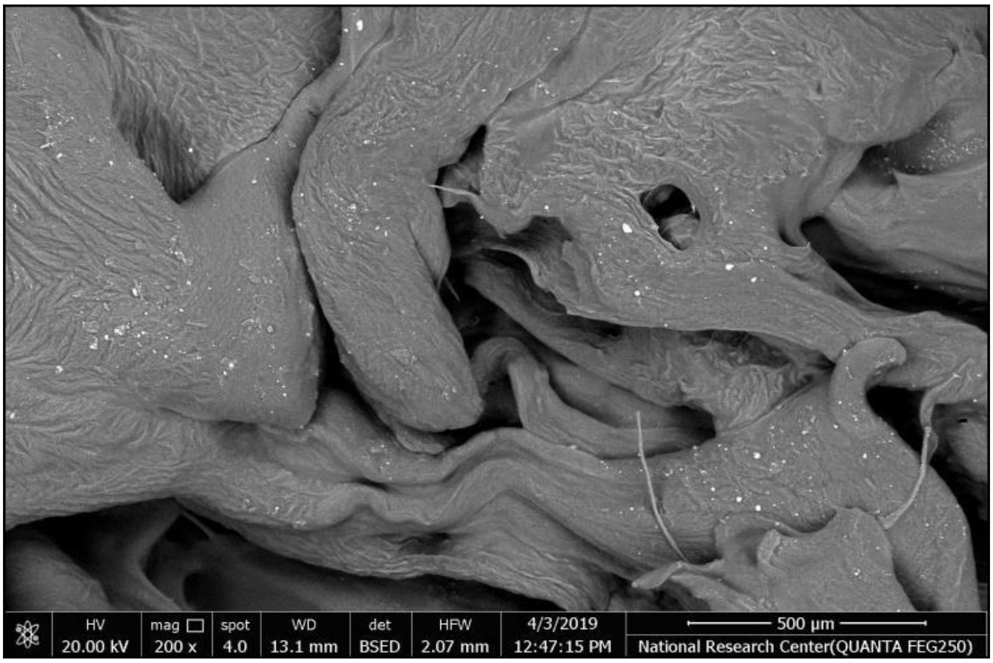

Figure S1. Transmission electron image of CRV nanosponge.

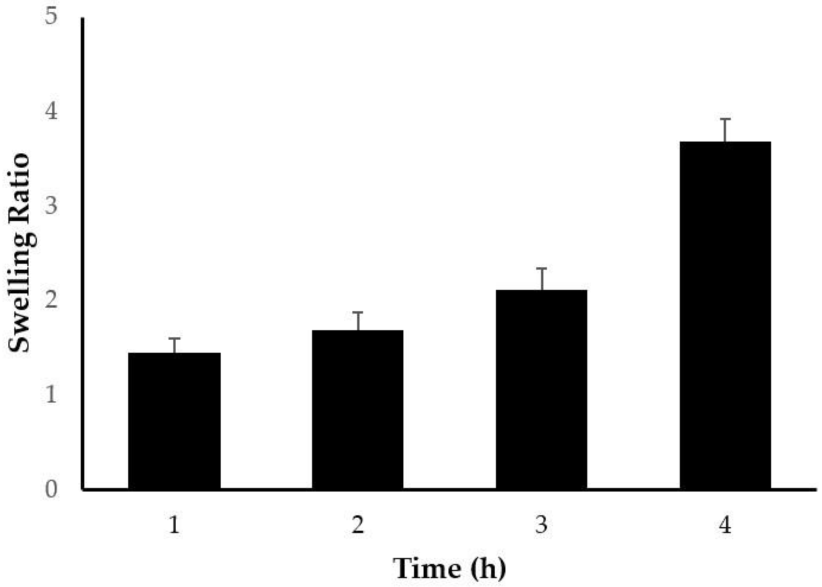

Figure S2. Swelling of CRV nanosponge.

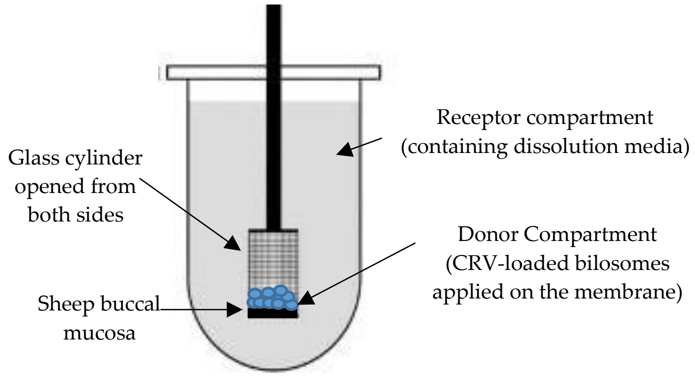

Figure S3. Cartoon depicting the apparatus utilized in *ex-vivo* permeation study of the optimized CRV-loaded bilosomes from Sheep buccal mucosa.

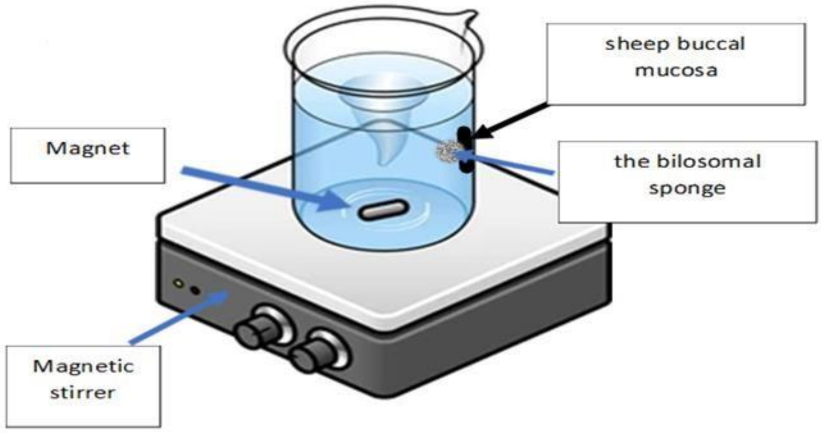

Figure S4. Cartoon depicting the apparatus utilized in measuring the ex-vivo mucoadhesion time of the formulated CRV nanosponge.
